# Conditioning of Rainbow Trout (*Oncorhynchus mykiss*) Broodstock in a High-Altitude Recirculating Aquaculture System: First Spawning at 3000 m.a.s.l. in Northern Chile

**DOI:** 10.3390/ani15111506

**Published:** 2025-05-22

**Authors:** Renzo Pepe-Victoriano, Piera Pepe-Vargas, Jordan I. Huanacuni, Héctor Aravena-Ambrosetti, Germán Olivares-Cantillano, Felipe Méndez-Abarca, Sheda Méndez, Luis Espinoza-Ramos

**Affiliations:** 1 Área de Biología Marina y Acuicultura, Facultad de Recursos Naturales Renovables, Universidad Arturo Prat, Iquique 101000, Chile; piera.pepe.vargas@gmail.com (P.P.-V.); jordan.92ihp@gmail.com (J.I.H.); hector.haravena.ambrosetti@gmail.com (H.A.-A.); fmendezabarca@gmail.com (F.M.-A.); smendez@estudiantesunap.cl (S.M.); 2 Núcleo de Investigación Aplicada e Innovación en Ciencias Biológicas, Facultad de Recursos Naturales Renovables, Universidad Arturo Prat, Iquique 1110939, Chile; 3 Programa de Magíster en Acuicultura, Mención Cultivo de Recursos Hidrobiológico y Mención Acuaponía, Facultad de Recursos Naturales Renovables, Universidad Arturo Prat, Iquique 1110939, Chile; 4 Finfish Aquaculture Sociedad Anónima Cerrada, Tacna 23004, Peru; 5 Piscicultura Río Blanco, Pontificia Universidad Católica de Valparaíso, Valparaíso 2340025, Chile; german.olivares@pucv.cl; 6 Departamento de Ingeniería Pesquera, Universidad Nacional de Moquegua (UNAM), Ilo 18601, Peru; 7 Escuela de Ingeniería Pesquera, Universidad Nacional Jorge Basadre Grohmann, Tacna 23004, Peru; lespinozar@unjbg.edu.pe

**Keywords:** aquaculture diversification, rainbow trout adaptability, RAS, reproduction, foothill aquaculture, photovoltaic system

## Abstract

This study monitored the conditioning of rainbow trout (*Oncorhynchus mykiss*) broodstock in a closed culture system at 3000 m above sea level (m.a.s.l.) to evaluate the feasibility of this culture method and assess the first spawning of the species under these conditions as an alternative production route for the communities present in the foothills of Cordillera de los Andes, northern Chile. A total of 120 specimens with an initial weight of 170 g were used. They were maintained in a recirculation aquaculture system (RAS) composed of protected circular tanks, decanters, a biofilter, and oxygenation equipment. Fish growth was monitored through parameters such as specific growth rate (SGR), feed conversion ratio (FCR) and percent weight growth (PWG), parameters that were maintained within those reported by other authors for this species. The first successful spawning occurred after 12 months of broodstock conditioning, yielding 8570 eggs, of which 6569 hatched successfully. The success of this conditioning and spawning not only broadens the understanding of adaptability to high-altitude environments, but also provides evidence for the potential diversification of productive activities in foothill areas through aquaculture.

## Simple Summary

This study assessed rainbow trout (*Oncorhynchus mykiss*) aquaculture at 3000 m above sea level in northern Chile’s Andes foothills as an alternative production method for local communities. A total of 120 fish were kept in a recirculating aquaculture system (RAS) with circular tanks, decanters, biofilters, and oxygenation. Growth parameters, including specific growth rate (SGR), feed conversion ratio (FCR), and percent weight growth (PWG), were monitored and found to be within expected ranges. After 12 months of broodstock conditioning, successful spawning occurred, yielding 8570 eggs, with 6569 hatching. This success highlights the potential for aquaculture in high-altitude areas.

## 1. Introduction

The growing global demand for animal protein, along with a shift toward healthier, lower-fat diets—especially in developed countries—has positioned aquaculture as a key solution to meet nutritional needs sustainably. In this regard, the Food and Agriculture Organization of the United Nations (FAO) has consistently promoted aquaculture as a strategic sector to ensure long-term protein supply at the local and global scales [1,2].

Chile is an important aquaculture producer, with edible fish among its most valuable products in both aquaculture and capture fisheries [2]. Notably, rainbow trout (*Oncorhynchus mykiss*), a non-native salmonid species in the region, has been extensively cultivated in the country [3]. However, production has been in steady decline for several years. Nearly two decades ago, annual production exceeded 200,000 tons, yet by 2023, it had fallen to just 44,288 tons, marking the lowest level recorded since 2012 [4].

In northern Chile, especially in the Arica and Parinacota Region, the decline of traditional rural activities has led to depopulation and economic stagnation. In this context, land-based freshwater aquaculture has been proposed as a sustainable alternative to revitalize local economies and improve food security in high-altitude areas [5,6,7,8]. The reuse of water for irrigation and the availability of infrastructure and microclimates suitable for fish farming support its implementation.

One of the main challenges for aquaculture in arid regions—such as northern Chile—is the scarcity of water resources [9,10]. However, the implementation of closed recirculating aquaculture systems (RASs) represents a viable alternative in pre-Andean areas. These systems can recycle 90% to 99% of the water used [10], as it undergoes treatment processes that allow its reuse in cultivation. This approach reduces water and energy consumption, while also minimizing nutrient discharge into the environment [11,12]. RASs also require less space and offer precise environmental control, enabling intensive production with high growth rates and yields [13].

From a management perspective, RASs allow for the precise regulation of key environmental parameters, which promotes faster growth and more efficient feed conversion by reducing stress on cultured organisms. Water temperature—an essential factor for poikilothermic species such as fish—can be regulated more effectively in RAS compared to open-flow systems [12]. Furthermore, increasing pollution in natural aquatic environments—particularly from organic contaminants and heavy metals—can adversely impact fish health and compromise production performance [14], making land-based, controlled systems a safer and more sustainable alternative.

In salmonid farming, as in other aquaculture species, the development and management of broodstock is fundamental for egg production, both in terms of quantity and quality. The quality of future larvae depends on that of the eggs, which in turn is determined by the health and management of broodstock [15,16]. Proper handling and conditioning of broodstock is therefore essential, especially if aquaculture is to be promoted as a diversification strategy in regions such as northern Chile.

Rainbow trout, a salmonid species of the genus *Oncorhynchus*, is among the most widely cultivated fish species worldwide due to its resilience and ease of rearing [17,18]. It tolerates a broad temperature range (0–25 °C), with optimal health maintained between 10 and 14 °C. For achieving good growth rates under optimal water quality, temperatures between 15 and 20 °C are preferred [19]. This species exhibits high physiological plasticity, allowing it to adapt to diverse environments, including high-altitude conditions. However, aquaculture at high elevations poses significant physiological challenges, primarily due to reduced atmospheric pressure and lower oxygen availability [20]. Oxygen is essential for fish metabolism, growth, and reproduction, as it directly influences aerobic respiration and energy production [21,22]. At high altitudes, reduced oxygen concentrations can impair gill gas exchange, potentially lowering growth performance, reproductive capacity, and overall broodstock health [23]. Nevertheless, studies suggest that fish raised at high altitudes can develop compensatory mechanisms, such as increased gill surface area, higher hemoglobin oxygen affinity, and metabolic adjustments to optimize oxygen uptake and use [24].

Despite the importance of these mechanisms, few international studies have examined the performance of O. mykiss in high-altitude aquaculture systems. For instance, Hernández-Gallegos [25], in a study conducted at approximately 2940 m a.s.l. in Mexico, reported variations in physiological stress levels based on site-specific conditions and management practices. Their results highlight the need for targeted management strategies to support rainbow trout adaptation to hypoxia, temperature fluctuations, and solar radiation. These findings are particularly relevant for evaluating the feasibility and challenges of trout farming in high-altitude regions such as the Chilean Altiplano, where biological and production data remain limited.

The aim of this study was to monitor and evaluate the conditioning and first spawning of rainbow trout broodstock in a recirculating aquaculture system (RAS) located in the foothills of northern Chile.

## 2. Materials and Methods

### 2.1. Study Area

**Figure 1:**
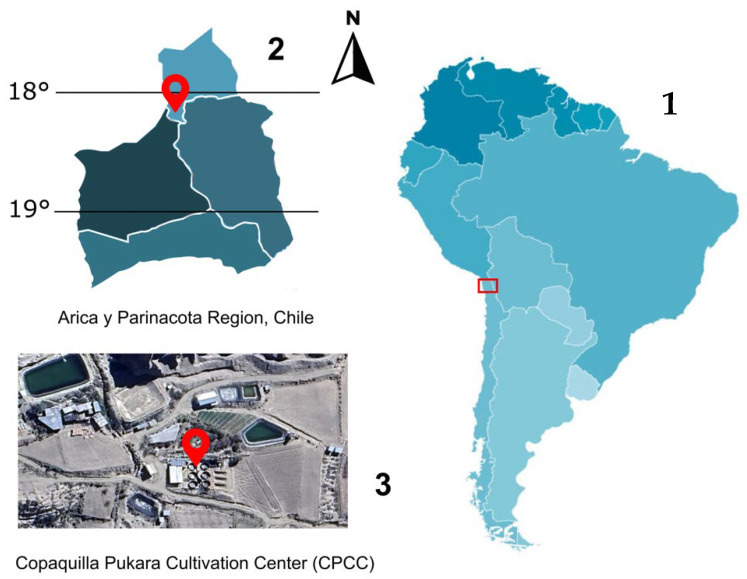
Geographical location of the Copaquilla Pukara Culture Center (CPCC) in the Arica and Parinacota Region, Chile. The figure includes (1) a map of South America with the region highlighted in red (**right**), (2) a detailed map of the Arica and Parinacota Region indicating the location of the CPCC (**upper left**), and (3) a satellite image of the cultivation center (**lower left**).

Conditioning and spawning of rainbow trout were conducted at the Copaquilla Pukara Culture Center (CPCC) (18°23′43″ S, 69°37′58″ W), located in the foothills of northern Chile, approximately 90 km inland from the city of Arica, at an altitude of 3000 m.a.s.l. (Figure 1).

### 2.2. Recirculating Aquaculture System (RAS) Setup

**Figure 2:**
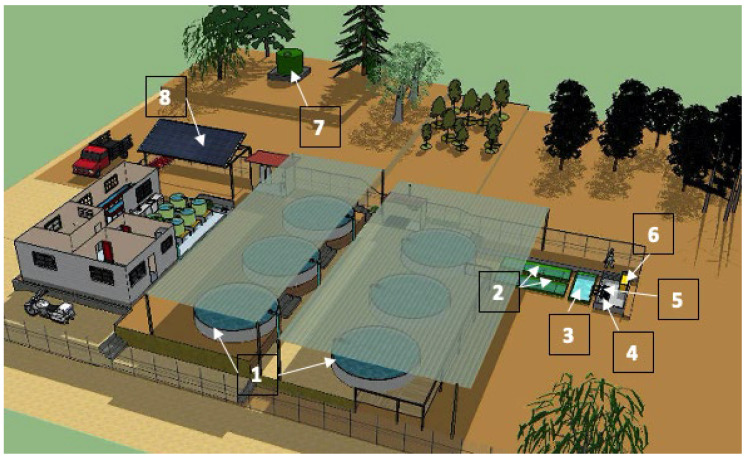
Schematic of the RAS used at the CPCC. The system includes (1) rearing tanks, (2) sedimentation tanks for solid removal, (3) biofilter tanks for water purification, (4) water circulation pumps, (5) aeration blower, (6) oxygen generator, (7) header tank for water reconditioning, and (8) photovoltaic panels for energy supply.

To cultivate potential rainbow trout broodstock, a recirculating aquaculture system (RAS) was implemented at the CPCC (Figure 2). The system consisted of six outdoor circular Australian-type tanks covered by a mesh roof to protect the fish from direct sunlight. Each tank featured a central drainage system and hydraulic connections for water supply and aeration. The system also included underground fiberglass tanks: two sedimentation tanks and one biofilter tank, in addition to a header tank for water reconditioning. Equipment included two water suction pumps (Reggio, model SM 150), a high-pressure blower (Sweetwater, 1.5 hp), and an oxygen generator (Oxiti, 8 LPM).

### 2.3. Water Quality Monitoring

Water used in the system originated from an underground source with low particulate content, making it suitable for aquaculture. However, it was oxygen- and nutrient-poor, requiring pre-oxygenation. Water was first stored in a tank and subsequently pumped to a 10 m^3^ header tank situated approximately 10 m above ground level. From there, water was allowed to fall freely, enhancing aeration through mechanical means.

Water quality was monitored at the system inlet, sedimentation tanks, and the header tank. Ammonium and nitrate were measured biweekly using a compact multi-parameter photometer (Hanna, model HI83303). When nitrate levels were elevated, up to 40% of the system water volume was replaced. Temperature and dissolved oxygen were measured three times daily (08:00, 13:00, and 18:00) using a portable oxygen meter (YSI, model I55).

### 2.4. Photovoltaic System

A photovoltaic (PV) system (Figure 2) consisting of 16 panels (250 W each), a Studer XTH 8000 inverter, a Track VT-80 charge controller, and 12 OPzS 2 V/2900 Ah solar batteries was installed. Additional components were also included to complete the system. The PV system provided energy autonomy for 10–12 h daily, with supplemental power supplied by a generator. Energy availability varied with biomass density in the tanks. PV panels were cleaned weekly using a soft dry cloth to maintain efficiency.

### 2.5. Selection and Conditioning of Breeding Stock

Out of ~5000 trout cultured at the CPCC (originally sourced from the Río Blanco hatchery, Pontificia Universidad Católica de Valparaíso), 120 individuals (~170 g average weight) were selected based on size and weight. The main population was distributed among four tanks, while the selected fish were moved to a dedicated tank for broodstock conditioning.

The selected fish were fed six days a week at 1.0–1.2 kg/day, corresponding to 6% of tank biomass. Juveniles received a diet containing 50% protein, 15% lipids, and 12% carbohydrates to maximize growth and feed efficiency. Broodstock received feed with 40% protein, 15% lipids, and 22% carbohydrates to promote gonadal development and gamete quality. After seven months, when average fish weight approached 800 g, feeding was gradually reduced to 1.5% of biomass.

Monthly random samplings of 20–30% of each tank population (~24–36 fish) were conducted to record weight using a digital scale (Mocco, model V-1026). Fish were handled carefully without sedatives and quickly returned to the tanks to minimize stress.

Specific growth rate (SGR) corresponds to the measure of the percentage increase in body weight per day. This was calculated as per Ricker [26],

SGR = [(ln W_f_ − ln W_i_)/T] × 100 (1)

where W_i_ = initial weight; W_f_ = final weight; T = time in days fed.

Percent weight growth (PWG) is understood as the difference between the final biomass minus the initial biomass per 100 [26], calculated as

PWG = [(W_f_ − W_i_)/Wi] × 100 (2)

where W_i_ = initial weight; W_f_ = final weight.

Feed conversion ratio (FCR) corresponds to an indicator that expresses weight gain of a cultured organism in relation to the weight of feed used. It was calculated by the following formula:

FCR = FG/AWG(3)

where FG = feed given (Kg); AWG = animal weight gain (Kg).

Survival rate was determined by recording daily mortalities per tank. This is expressed as percentage survival (%S) [26] as follows:

% S = (n_f_/n_i_) × 100 (4)

where n_i_ = initial number of individuals; n_f_ = final number of individuals.

### 2.6. Spawning

After 12 months of broodstock conditioning, the first spawning involved four females and twelve males, selected based on sexual dimorphism related to maturation [27]. Gametes were obtained by manual abdominal stripping without anesthesia to reduce stress. The male-to-female ratio was 3:1 across four spawning events conducted on the same day.

Eggs were collected dry in stainless-steel containers, fertilized with milt, and washed with filtered system water until it ran clear. Fertilized eggs were incubated in darkness at 10 °C in four trays placed within a pan inside the incubation room. During the first five days post-fertilization, dead eggs—recognized by their pale coloration—were removed via siphoning. Hatching occurred 26–28 days post-fertilization.

To determine fecundity, each female’s weight was recorded, and her egg count was determined manually in a controlled laboratory setting. Fecundity was calculated as follows:

Egg production rate = Total number of eggs/Female weight (kg)

### 2.7. Data Analyses

All statistical analyses were performed using RStudio (version 2024.09.0+375; RStudio, Inc. Washington, United State, source: Peru). Normality and homogeneity of variances were tested using the Anderson–Darling and Bartlett’s tests, respectively. Pearson’s correlation was applied to assess associations among variables. A *p*-value < 0.05 was considered statistically significant [28]. Graphs were created with the ggplot2 package from RStudio, and results are presented as mean ± standard deviation (SD).

## 3. Results

### 3.1. Water Quality Monitoring

Monthly average values of temperature and dissolved oxygen are shown in Table 1. A decrease in temperature was recorded during the colder months (April to October), with values dropping below 7.4 ± 0.40 °C. In contrast, temperatures increased during the warmer months (November to March), reaching up to 18 ± 0.39 °C between January and March.

Dissolved oxygen in the tanks showed an inverse seasonal pattern, with higher levels during the colder months (up to 7 ± 0.38 mg L^−1^) and lower levels during warmer periods (as low as 4.9 ± 0.23 mg L^−1^), likely due to increased fish metabolism and oxygen consumption at higher temperatures.

Table 1 presents the concentrations of ammonium and nitrate during the broodstock conditioning period. Peaks in nitrate levels triggered water changes, as reflected in the subsequent declines. In contrast, ammonium levels remained relatively stable, ranging from 0.2 to 0.7 mg L^−1^, due to effective control by the biofilter. These concentrations did not pose a risk to fish health.

### 3.2. Feeding and Growth

**Figure 3:**
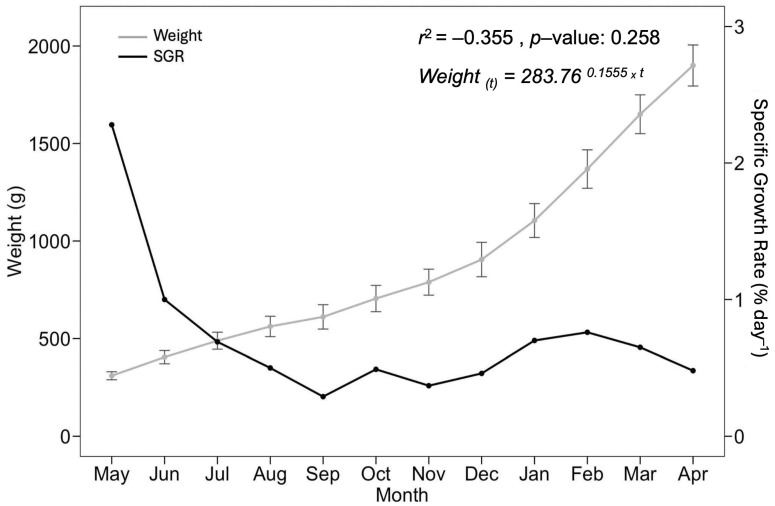
Growth progression of rainbow trout broodstock over 12 months, showing trends in average weight (g) and specific growth rate (SGR) during the conditioning phase.

Figure 3 shows the weight increase in broodstock over the 12-month conditioning period, evidencing a steady growth trend that resulted in an average weight exceeding 1900 g (r^2^ = –0.35). SGR, FCR, and other performance metrics are summarized in Table 2. The SGR values are also plotted in Figure 3. 

### 3.3. Spawning

Four spawning events were conducted using different male and female combinations. Table 3 summarizes the reproductive performance for each event. Variability was observed in the proportion of removed and hatched eggs across spawnings, without a consistent pattern.

### 3.4. Statistical Evaluation of Results

**Figure 4:**
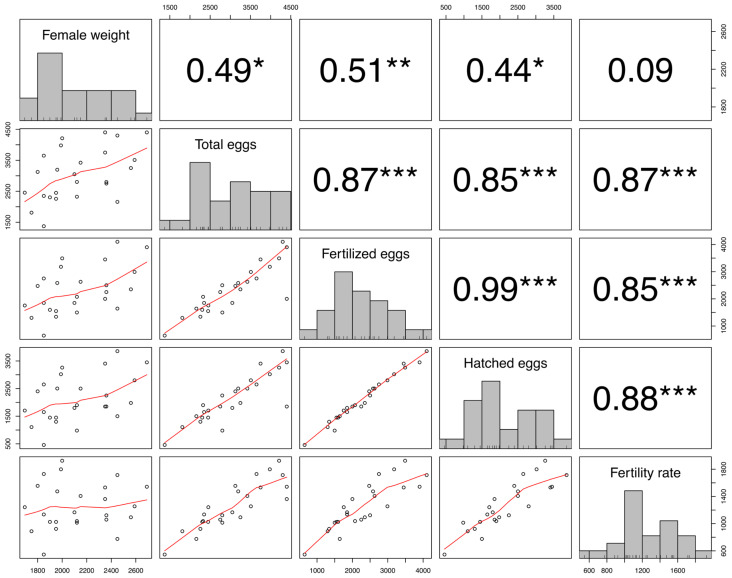
Scatter plots showing distributions and correlations between female weight, total eggs, fertilized eggs, hatched eggs, and fertility rate in *Oncorhynchus mykiss* broodstock. Significance thresholds: *p* < 0.05 (*), *p* < 0.01 (**), and *p* < 0.001 (***).

Figure 4 displays the correlations among the main reproductive and biometric variables. A nearly perfect correlation was observed between the total number of eggs per spawning and the number of fertilized eggs (r^2^ = 0.99). Fertilized and hatched eggs also showed a strong positive correlation (r^2^ = 0.88). Similarly, the fertility rate correlated positively with total eggs (r^2^ = 0.87), fertilized eggs (r^2^ = 0.85), and hatched eggs (r^2^ = 0.87).

Moderate positive correlations were found between female weight and total eggs (r^2^ = 0.49) and fertilized eggs (r^2^ = 0.51). However, no significant correlation was observed between female weight and fertility rate (r^2^ = 0.086).

## 4. Discussion

Rainbow trout farming at high altitudes (2000–3000 m.a.s.l.) is well established in Latin American countries such as Colombia, Ecuador, Bolivia, and Peru [29,30,31,32,33], where it occurs in both tank systems and lake cages. In contrast, salmonid aquaculture in Chile has historically focused on southern coastal areas, leaving the pre-mountain and mountain zones of northern Chile (18° S to 21° S) underutilized despite suitable environmental conditions.

Historical efforts to introduce rainbow trout farming in northern Chile date back to 1993–1995 in localities such as Caquena, Ancolacane, Cosapilla, and Colpita, aiming to support Aymara communities and reduce rural–urban migration. These initiatives included incubation and fry units, hydraulic networks, and later, infrastructure upgrades like settling tanks [34]. However, no current operations in the region match the production scale or technological level achieved in this study.

### 4.1. Water Quality

Ensuring specific water quality conditions is essential for successful fish farming. Monitoring key variables allows optimal growth and survival, especially in systems like ours that rely on high-quality groundwater. This source, with minimal turbidity, requires no treatment beyond gravity-driven aeration from the head tank (Figure 2), eliminating the need for mechanical filtration.

Rainbow trout (*Oncorhynchus mykiss*) are ectothermic; their growth, feed efficiency, disease resistance, and reproduction depend directly on water temperature [35]. Although they can tolerate a range from 0 to 25 °C [36], optimal development occurs between 9 and 17 °C [37,38,39], particularly between 13 and 18 °C for fattening. Our system’s temperatures consistently fell within these ranges, with monthly averages showing seasonal variation: colder months (April to October) had temperatures dropping below 7.4 ± 0.40 °C, while warmer months (November to March) reached up to 18 ± 0.39 °C (Tabla1).

Dissolved oxygen exhibited an inverse seasonal pattern, with higher levels during colder months (up to 7 ± 0.38 mg L^−1^) and lower levels in warmer months (as low as 4.9 ± 0.23 mg L^−1^), likely due to increased fish metabolism and oxygen consumption at higher temperatures. Although some low oxygen values were recorded, no mortalities occurred, probably due to compensatory physiological responses such as enhanced gill gas exchange [40]. Nevertheless, maintaining dissolved oxygen above critical thresholds remains crucial for sustained growth.

Water quality management in our recirculating aquaculture system (RAS) also requires tight control of nitrogenous wastes. Table 1 presents ammonium and nitrate concentrations during broodstock conditioning. Peaks in nitrate triggered water changes, resulting in subsequent decreases, reflecting active system maintenance. Ammonium levels remained relatively stable, ranging from 0.2 to 0.7 mg L^−1^, controlled effectively by the biofilter. Although these values occasionally surpassed the conservative threshold of <0.012 mg L^−1^ recommended by Camacho [41], no significant negative impacts on fish health or mortality were observed, indicating trout resilience.

Given the sensitivity of rainbow trout to ammonia toxicity [42,43], maintaining low ammonium concentrations is critical. This control mitigates the risk of toxic un-ionized ammonia formation, which can increase tenfold with just a one-unit rise in pH [44], and is exacerbated at higher temperatures due to increased NH₃ volatilization [45,46]. Fish density also influences nitrogen accumulation, affecting growth, immunity, and gill health [44,45]. Our integrated water quality management system—including temperature regulation, oxygen monitoring, and biofiltration—supports optimal fish performance and system sustainability, particularly in the challenging environmental context of high-altitude aquaculture.

### 4.2. Feeding and Growth

At the CCPC, the feed conversion ratio (FCR) was 1.3, indicating that 1.3 kg of feed was required for the fish to gain 1 kg of body weight. Although the ideal FCR is close to 1, this result still reflects efficient feed use under the studied conditions [47,48]. Environmental stressors such as heavy altiplanic rains falling on the protective mesh and thunder may have temporarily affected appetite; however, this was not reflected in the FCR, and fish growth was acceptable (Figure 3).

Yapachiura-Saico et al. [47] reported a specific growth rate (SGR) of 1.0 after short-term fasting, while Huanca [49] and Morales [48] documented values between 1.0 and 1.35 under continuous feeding. In our study, with feeding six days per week, the average SGR was 0.77. Although lower than previous reports, this slower growth can be attributed to high-altitude environmental stress, including fluctuating temperatures, rainfall, and noise.

Despite these limitations, growth performance remained satisfactory, supported by a stable FCR and the growth trend observed in early months (Figure 3), likely linked to the higher metabolic rates of juvenile fish [50]. Regular feeding also promotes uniformity in size and weight, which is essential for production efficiency [51].

The percent weight growth (PWG) approached 1000% (Table 2), surpassing previously reported PWG values for this species—ranging from 125% to 623% [52,53]—and even for other fish such as turbot (374–668%) [51] and snook (323–514%) [54]. These values are comparable to PWG values in rainbow trout from Rosales-Marín [55] and Perdomo et al. [56], who reported up to 1157.1%.

Survival rates remained high throughout the culture period (Table 2), confirming that the conditions at CCPC were favorable for broodstock development. These results demonstrate the adaptability of *Oncorhynchus mykiss* to the Chilean Altiplano (~3000 m a.s.l.), consistent with other high-altitude studies. For instance, Hernández-Gallegos [25] in Mexico (~2940 m a.s.l.) emphasized the role of farm practices in mitigating stress, while Mouillet et al. [57] documented ecological effects of trout in natural systems.

Unlike those studies, ours focused on intensive farming using a photovoltaic-supported recirculating aquaculture system (RAS). Despite environmental constraints such as hypoxia and temperature fluctuations, the system supported positive zootechnical outcomes. Notably, our FCR (1.3) is comparable to values reported under temperature-controlled conditions in Finland (1.2–1.6) [58], suggesting that efficient feed use can be achieved even without artificial thermal regulation in extreme environments.

### 4.3. Broodstock and Spawning

The conditioning of fish broodstock should start with early selection based on growth parameters from hatching, focusing on individuals with rapid growth rates that align with expected species values [51,59]. Genetic characteristics, not just phenotypic traits, should also be considered in selecting potential breeders [59,60]. In our study, we used only phenotypic traits for conditioning juvenile trout broodstock averaging 170 g. While genetic selection could improve precision, it was not feasible in our low-cost, small-scale setting at over 3000 m above sea level, where access to genetic tools is limited. We recommend incorporating genetic criteria in future research as resources and technical capacity in high-altitude aquaculture develop.

Reproductive success in captivity is influenced by both internal factors (e.g., genetic, egg composition, broodstock quality) and external factors (e.g., water temperature, salinity, nutrition) [61]. Achieving high reproductive yields is challenging for producers across scales, as interactions between these factors vary among individuals. The small sample size (*n* = 4) in our study, typical for initiatives supporting local pre-Andean Indigenous communities, may limit generalizability. Future studies involving greater numbers of broodstock are needed to strengthen the results.

The temperature in our conditioning system averaged 15.1 °C, with fluctuations between 8.9 °C and 20.5 °C, without temperature regulation due to outdoor conditions. In contrast, Vilcherrez and Pardo [62] maintained breeders in closed spaces, with more stable temperatures (8.8 °C to 9.2 °C), which has been shown to reduce stress and improve spawning and broodstock survival [63,64]. Despite the high temperature variation in our study, we observed zero mortality, unlike Vilcherrez and Pardo [62], who reported mortality rates below 20%. Our conditioning experiment achieved 98.33% survival, suggesting that successful outcomes are possible even under these challenging conditions.

Broodstock age and prior spawning history can impact spawning quality [65]. For example, Vilcherrez et al. [62] worked with two-year-old rainbow trout, yielding an average of 5392 eggs per female. In our study, we observed 2135 eggs per female, a value lower than reported in Costa Rica [37] and by Vilcherrez et al. [62]. This could be due to our females’ first spawning cycle, where primiparous females typically produce fewer eggs [66]. Additionally, no selection was applied beyond initial conditioning.

Fertilization rates, often used as indicators of egg quality [67], were favorable in our study, with a mortality rate of 23.3%, much lower than the 46.2% reported by Vilcherrez and Pardo [62]. However, the fecundity rate (Table 3) was lower than the 2200 eggs per kg female reported for the species [68]. This could be explained by our broodstock being in their first spawning cycle, as demonstrated in other studies [69,70]. The challenging environmental conditions at high altitude may have further affected reproductive performance.

During sexual maturation, lipid mobilization is crucial for ovarian development but can compromise egg quality [71]. The partial replacement of fish oil with vegetable oils in broodstock diets can alter fatty acid composition in eggs, potentially affecting fertilization and embryonic development [72]. Antioxidant supplementation, such as Haematococcus pluvialis, has shown potential in improving egg quality [73]. These factors underscore the importance of managing nutrition and environmental stress to optimize broodstock reproductive success.

Our results show a strong correlation (r^2^ = 0.99) between total eggs and fertilized eggs, and a positive correlation (r^2^ = 0.88) between fertilized and hatched eggs, indicating optimal fertilization conditions. The correlation between female weight and egg count was moderate (r^2^ = 0.49), suggesting that other factors like age and body condition are significant. The correlation between female weight and fertility rate was non-significant (r^2^ = 0.086), aligning with studies indicating that factors beyond size, such as oocyte quality and environmental conditions, are critical to fertility [74].

Although dissolved oxygen and ammonia were addressed from a physicochemical perspective, it is important to consider their physiological implications on fish reproduction and stress responses. Studies have shown that ammonia exposure, especially when combined with other stressors, can reduce reproductive performance and increase oxidative stress [75]. Furthermore, evidence from fish models suggests sex-specific responses to ammonia, highlighting the importance of considering sexual dimorphism when assessing physiological impacts [76]. These insights are relevant for improving broodstock management and water quality strategies in intensive aquaculture systems.

### 4.4. Photovoltaic System

The RAS at Copaquilla, located approximately 3000 m above sea level, incorporates photovoltaic (PV) technology to address the specific challenges of high-altitude aquaculture. In remote areas with limited access to the power grid and subject to extreme climatic conditions, stable and autonomous energy sources are essential to ensure continuous water recirculation and temperature regulation—both critical for the optimal performance of *Oncorhynchus mykiss*.

Although no operational data on the PV system were collected in this study, its implementation reflects a novel and practical solution for sustainable aquaculture in isolated mountain environments. The use of solar energy reduces dependence on fossil fuels, minimizes carbon emissions, and promotes energy resilience—key factors for the viability of remote aquaculture ventures.

A comparable initiative was developed in Camarones [77], a locality also in the Arica y Parinacota Region, where a larger and more advanced solar-powered RAS was established to farm river shrimp and rainbow trout. That system additionally integrates solar water treatment to reduce arsenic and applies circular economy principles to reuse aquaculture residues in agriculture. While the scale and complexity of the Camarones system differ from ours, both cases highlight the growing relevance of PV-based aquaculture in northern Chile and demonstrate how renewable energy can foster sustainable development in environmentally and logistically challenging areas.

## 5. Conclusions

This study confirms the feasibility of conditioning rainbow trout (*Oncorhynchus mykiss*) broodstock at 3000 m a.s.l., demonstrating their capacity to adapt to high-altitude environments. The results highlight the importance of water quality control and other key factors to ensure successful reproduction and fish survival under these conditions.

Moreover, the findings reinforce the potential for aquaculture diversification in highland areas, promoting a production model that supports regional development through efficient and responsible resource use.

Unlike the dominant salmon industry—which concentrates most aquaculture research in Chile—this study offers concrete evidence supporting small-scale aquaculture, generating knowledge that is applicable and adapted to local producers. In this way, it opens the door to future research that considers the specific needs of more sustainable and locally relevant initiatives.

## Figures and Tables

**Table 1:** Physicochemical parameters of water during the evaluation period of *Oncorhynchus mykiss* (2015–2016).

Year	Month	Temperature (°C)	Dissolved Oxygen (mg/L)	Ammonia (mg/L)	Nitrate (mg/L)
2015	May	10.0 ± 0.9	5.8 ± 0.2	0.56	1.3
	June	7.4 ± 0.4	5.4 ± 0.4	0.31	0.69
	July	7.5 ± 0.4	6.0 ± 0.8	0.52	0.65
	August	8.0 ± 0.3	7.0 ± 0.4	0.32	0.52
	September	10.0 ± 0.7	5.0 ± 0.4	0.59	0.68
	October	13.0 ± 0.8	6.0 ± 0.4	0.65	1.45
	November	16.2 ± 0.5	5.1 ± 0.6	0.25	0.65
	December	17.0 ± 0.8	5.0 ± 0.4	0.48	0.89
2016	January	18.0 ± 0.4	5.3 ± 0.4	0.36	0.74
	February	18.0 ± 0.3	4.9 ± 0.2	0.25	0.69
	March	17.4 ± 0.5	5.3 ± 0.3	0.65	1.58
	April	16.0 ± 0.4	5.0 ± 0.4	0.36	0.68
Media ± DE		13.2 ± 4.4	5.5 ± 0.6	0.4 ± 0.2	0.9 ± 0.4
Shapiro–Wilk (*p*-value)		0.031	0.024	0.168	0.004
Correlation (r^2^)		0.818	−0.632	−0.0667	0.200
Spearman (*p*-value)		0.001	0.027	0.837	0.532

**Table 2:** Growth performance metrics of rainbow trout broodstock in the recirculating aquaculture system (RAS).

Variable	Broodstock Tank
Food provided (kg)	271
Initial biomass (kg)	20.4
Final biomass (kg)	224.2
Increase in weight (kg)	203.8
Initial density (kg m^3^)	0.51
Final density (kg m^3^)	5.61
Initial number of fishes	120
Final number of fishes	118
Feed conversion ratio (FCR)	1.33
Specific growth rate (SGR)	0.77
Percent weight growth (PWG))	999.02
Survival rate (%)	98.33

**Table 3:** Reproductive performance of rainbow trout broodstock during four spawning events.

	1st Spawning	2nd Spawning	3rd Spawning	4th Spawning
No. fertilized eggs	1555	1010	3025	2890
No. hatched eggs	1106	459	2501	2503
No. eliminated eggs	449	551	524	387
No. males	3	3	3	3
No. females	1	1	1	1
Female weight	1750	1850	2150	1960
Fertility rate	888	546	1407	1474

## Data Availability

The data presented in this study are available on request from the corresponding author. The data are not publicly available for privacy reasons.
